# Low-molecular-weight heparin in the prevention of unexplained recurrent miscarriage: a systematic review and meta-analysis

**DOI:** 10.1038/s41598-024-62949-5

**Published:** 2024-06-19

**Authors:** Margherita Scarrone, Noemi Salmeri, Giovanni Buzzaccarini, Valentina Canti, Federica Pasi, Enrico Papaleo, Patrizia Rovere-Querini, Massimo Candiani, Alessandra Alteri, Andrea Busnelli, Valeria Stella Vanni

**Affiliations:** 1 Università Vita-Salute San Raffaele, Via Olgettina 60, 20132 Milan, Italy; 2 Department of Obstetrics and Gynecology, IRCCS San Raffaele Scientific Institute, Milan, Italy; 3 Division of Immunology, Transplantation and Infectious Diseases, IRCCS San Raffaele Scientific Institute, Via Olgettina 60, 20132 Milan, Italy; 4 Reproductive Sciences Laboratory, IRCCS San Raffaele Scientific Institute, Via Olgettina 60, 20132 Milan, Italy; 5 Department of Biomedical Sciences, Humanitas University, Pieve Emanuele-Milan, Via Rita Levi Montalcini, 4, 20090 Rozzano, Milano Italy; 6 Division of Gynecology and Reproductive Medicine, Department of Gynecology, Fertility Center, IRCCS Humanitas Research Hospital, Via Rita Levi Montalcini, 4, 20090 Rozzano, Milano Italy

**Keywords:** Autoimmunity, Coagulation system, Endocrine system and metabolic diseases

## Abstract

The etiology of recurrent pregnancy loss (RPL) is complex and multifactorial and in half of patients it remains unexplained (U-RPL). Recently, low-molecular-weight heparin (LMWH) has gained increasing relevance for its therapeutic potential. On this regard, the aim of this systematic review and meta-analysis is to analyze the efficacy of low molecular weight heparin (LMWH) from the beginning of pregnancy in terms of live birth rates (LBR) in U-RPL. Registered randomized controlled trials (RCTs) were included. We stratified findings based on relevant clinical factors including number of previous miscarriages, treatment type and control type. Intervention or exposure was defined as the administration of LMWH alone or in combination with low-dose aspirin (LDA). A total of 6 studies involving 1016 patients were included. The meta-analysis results showed that LMWH used in the treatment of U-RPL was not associated with an increase in LBR with a pooled OR of 1.01, a medium heterogeneity (26.42%) and no publication bias. Results of other sub-analyses according to country, treatment type, and control type showed no significant effect of LMWH on LBR in all subgroups, with a high heterogeneity. The results highlight a non-significant effect of LMWH in U-RPL on LBR based on moderate quality evidence.

**Registration number:** PROSPERO: (https://www.crd.york.ac.uk/prospero/display_record.php?ID=CRD42022326433).

## Introduction

No consensus has been reached on the definition of recurrent Pregnancy Loss (RPL), which can be defined as the repetitive loss of clinical pregnancies, either consecutive or not^1–3^. The American Society for Reproductive Medicine (ASRM) defines recurrent pregnancy loss as two or more failed clinical pregnancies documented by ultrasonography or histopathologic examination. This definition reflects a shift from previous criteria, which required three or more miscarriages^4^. On the other hand, the European Society of Human Reproduction and Embryology (ESHRE) has a similar approach to defining RPL, also identifying it as the loss of two or more pregnancies. ESHRE emphasizes the need for a thorough investigation after two losses, particularly in women aged over 35 years or with additional risk factors, to provide appropriate guidance and treatment without undue delay^5^. The lack of a consensus on its definition has consequences also on the estimated prevalence, which is around 1% when three miscarriages are considered^6^ and rises to 5% if we consider two miscarriages^7^. The etiology of RPL is complex and multifactorial: several etiologic factors have been linked to RPL including chromosomal and genetic abnormalities, congenital and acquired thrombophilia, endocrine disorders, autoimmune diseases and uterine anatomical abnormalities^8^. Even after extensive investigations, we are able to identify a cause of RPL in only half of the patients screened^9^. In the remaining cases, RPL remains unexplained, thus no targeted therapy can be used and only empirical therapies can be prescribed.

Indeed, often U-RPL is treated with progesterone administration in order to support the lutheal phase, with results that become significant after three or more previous miscarriages, or in the presence of first-trimester vaginal bleeding and a history of one or more previous miscarriage^10^. Other potential treatments are highlighted as add-ons and no evidence is still provided.

As a matter of fact, acceleration of high-quality evidence in this field has been described as an essential priority^11^. In the context of unexplained RPL (U-RPL), low molecular weight heparin (LMWH) is prescribed in up to half of patients (sistemare nuova) Ref.^12^, despite not being supported by current guidelines. Heparin exerts a range of non-anticoagulant actions that are highly relevant to embryo implantation and placental development^12^. Heparin exerts an immunomodulatory action by means of several molecular mechanisms including regulation of leukocytes’ function, inhibition of complement activation and proinflammatory cytokines production^13–15^, In the context of antiphopholipid syndrome, heparin may interfere with the binding of antiphospholipid antibodies through a mechanism of competitive inhibition^16^. Furthermore heparin may promote embryo implantation by increasing trophoblast proliferation^14,15^. Literature in the clinical setting has however provided non-conclusive results as to wether LMWH administration does indeed impact live-birth rates^17–23^. Discrepancy between studies’ findings is most likely due to (i) the lack of a unanimous definition; and (ii) the fact that many studies did not exclude from the U-RPL population under study RPL patients affected by any kind of thrombophilia^24–27^. Two recent meta-analyses^22,28^ based on randomized, controlled trials (RCTs) concluded that LMWH has no beneficial effects on U-RPL. However, some of the available published RCTs were excluded from such studies, either for use of low-dose aspirin (LDA) in the control group^19,29,30^ or in some cases for no clear reason^17^. Notably, a different meta-analysis on RCTs confirmed a beneficial effect of LMWH on miscarriage rates in women with U-RPL defined as 3 or more previous abortions^31^, but one of the included studies^32^ was subsequently retracted, and one study included also patients with thrombophilia.

The objective of our systematic review and meta-analysis is thus to properly assess whether LMWH administration has an impact on live birth rates in women affected by U-RPL. For this reason, we excluded patients affected by thrombophilia. In line with the comprehensive intent of this study, we have accounted for possible confounding factors through ad hoc sub-analyses.

## Materials and methods

This literature overview was reported according to the PRISMA guidelines for systematic reviews^33,34^ and the meta-analysis was conducted according to the MOOSE guidelines^35^. Since published de-identified data were used, this study was exempt from institutional review board approval. A protocol for this systematic review and meta-analysis has been registered at PROSPERO (https://www.crd.york.ac.uk/prospero/display_record.php?ID=CRD42022326433).

### Search strategy and selection criteria

We systematically searched PubMed, MEDLINE, Cochrane CENTRAL database, Embase and Scopus, from database inception to June 30th, 2023. Searches were limited to studies in humans and were conducted using the following terms: (recurrent miscarriage OR habitual abortion OR recurrent pregnancy loss OR recurrent abortion) AND (idiopathic OR unexplained) AND (heparin OR LMWH OR antithrombotic). Studies were included only if: (1) they included patients with U-RPL defined as either 2 or 3 previous first trimester miscarriages and whose complete RPL diagnostic work-up was negative; (2) the authors reported the live birth rate (LBR); (3) the treatment group received LMWH alone or in combination with LDA and the control group received placebo, other treatments or no treatment. Diagnostic work-up for RPL had to include exclusion of: congenital and acquired thrombophilia, thyroid abnormalities, congenital uterine malformations, parental karyotype anomalies. In order to comply with scientific integrity requirements^36^, unregistered RCT published after 2010 and articles whose authors have other retracted/fabricated studies as listed on http://retractiondatabase.org/ were excluded. All pertinent articles were retrieved and respective reference lists were systematically reviewed to identify additional reports for inclusion in the meta-analysis. Moreover, review articles and meta-analyses that focused on the efficacy of LMWH in the treatment of women affected by U-RPL were consulted and their reference lists searched for potential additional studies. No attempt was made to identify unpublished studies.

### Intervention

Any type of LMWH therapy either alone or in combination with LDA, with no limitations regarding type, dosage and duration.

### Selection of studies

All relevant literature including abstracts and full text articles were reviewed by three independent investigators (MS, NS, VSV). Studies were excluded if they were deemed irrelevant by all the three observers.

### Data extraction

Two authors (MS and NS) independently evaluated all articles and extrapolated the data on standardized forms. A final abstraction form was compiled from the two evaluation forms after a discussion with the remaining authors resolved any reviewer discrepancies. For every study, the following data were extracted: study characteristics (publication year, region, design of the study, study center), patients’ characteristics (number of subjects enrolled, age, BMI, obstetric history), LMWH treatment’s characteristics (type, dosage and duration), and primary outcomes (miscarriage rate and/or LBR).

### Outcome measure

The primary outcomes of this meta-analysis was LBR.

### Risk of bias evaluation

Three authors (MS, NS and VSV) independently assessed the included studies for risks of bias using the Newcastle–Ottawa Quality Assessment Scale (NOS) for cohort studies^37^ and the Cochrane ‘Risk of bias’ assessment tool 2 (RoB2) for RCTs^38^.

### Quality of evidence assessment

Two authors (GB and EP) also graded the quality of evidence using the Grading of Recommendations Assessment, Development and Evaluation (GRADE) approach^39^ and considering the main outcome (LBR). Quality of evidence was downgraded by one level for serious concerns and by two levels for very serious concerns for risk of bias, inconsistency, indirectness, imprecision and publication bias. For each of the two outcomes, a GRADE evaluation was presented. Moreover, a TRACT analysis was performed on the included articles^36^.

### Statistical analysis

Data from original articles were extracted and tabulated by two independent authors (MS, NS). The risk estimates for miscarriage and live birth were abstracted and tabulated by an independent reviewer (NS) in terms of odds ratios (OR) with 95% confidence intervals (CI). Log transformed study-specific effect sizes were pooled together to provide a weighted average of the study-specific effect sizes, with larger and more precise studies having greater weights. Heterogeneity of included studies was measured throughout the I^2^ statistics^40^, as a result of clinical, methodological and statistical heterogeneity. Random-effects models with DerSimonian–Laird method were performed for both the outcomes investigated (live birth and miscarriages) to pool together risk estimates of all the studies included in primary analysis. To explore the relationship between the estimated effect sizes and multiple study-level covariates (i.e., moderators), subgroup analysis were performed according to random or fixed-effects models according to heterogeneity of included studies^33^. Publication bias was explored by scattering study-specific effect sizes versus the measure of study precision. Funnel plot asymmetry was checked out according to Egger et al. (with evidence of asymmetry based on p-value < 0.1)^33,34^. STATA version 17 software (Stata Corp LLC, 2021, College Station, TX, USA) was used for all statistical analyses. A p-value < 0.05 was considered to be statistically significant.

## Results

### Study selection

**Figure 1:**
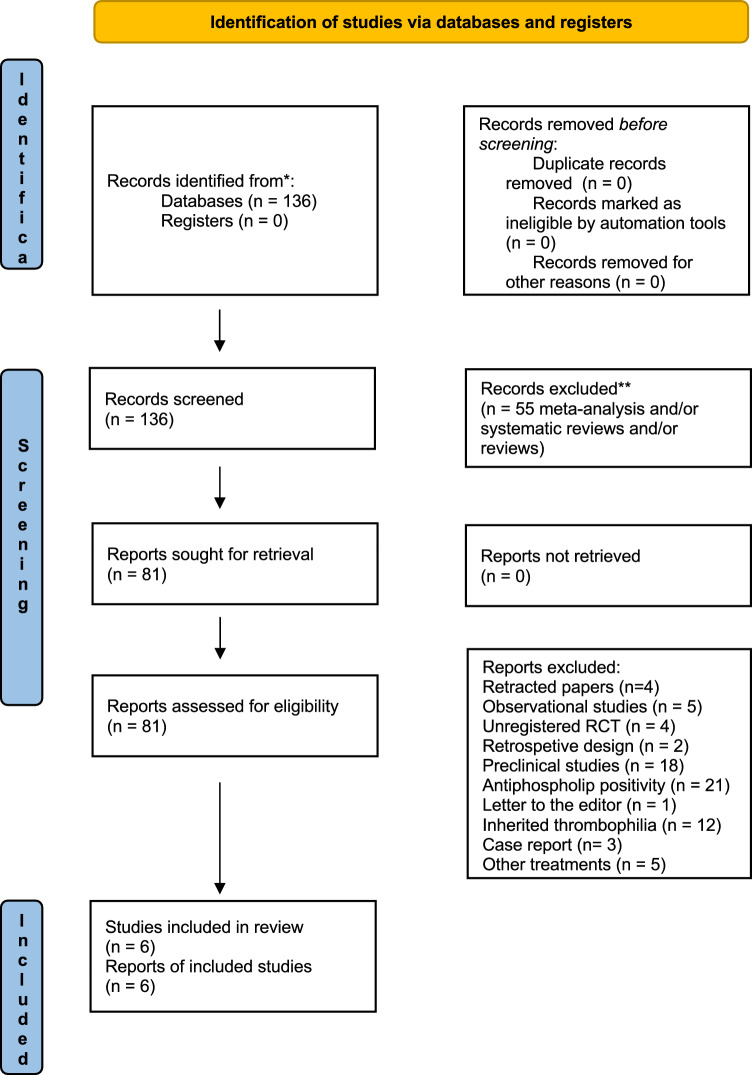
PRISMA 2020 flow diagram for the identification of studies included in the systematic review and meta-analysis.

There were 136 relevant articles identified through electronic databases. A flowchart of the literature search and study selection results is shown in Fig. 1. All titles and abstracts were screened for exclusion criteria. After screening of titles and abstracts we excluded n = 58 records being reviews and/or systematic reviews and/or meta-analyses. Then, 81 articles were screened reading the full text: n = 5 studies were excluded because of the observational design, n = 2 were excluded because of the retrospective design, n = 4 RCTs were excluded because of lack of registration, n = 18 were preclinical studies, in n = 21 studies patients were positive for antiphospholipid antibodies, one study was a Letter to the editor, n = 12 studies did not exclude patients with thrombophilia, n = 3 studies were case reports, in n = 5 studies patients were prescribed other treatments than LMWH, and one study was retracted^24^, n = 3 studies were excluded because of previous retracted papers by the same authors. Data relevant to the effect of LMWH therapy on MR and/or LBR in women with U-RPL were extracted from the remaining 6 articles including 1016 participants^17,19,21,29,30,41^.

### Study characteristics

A total of 518 participants with U-RPL received LMWH. Included patients were administered: (i) enoxaparin in five studies^17,19,29,30,41^, nadroparin in one study^21^. The 498 patients in the control group received either a placebo, other treatments, or no treatment. Characteristics of included studies are reported in Table 1. The LBR was reported in 6 out of 6 studies. Power calculation analysis finds that—with an expected 75% chance of a live-birth after 2 consecutive miscarriages—n = 1080 patients must be included in order to detect a significant 7% increase in this figure (i.e. expected live-birth rate 82%) with 80% power and 0.05 alpha-error. Our meta-analysis includes a total of n = 1111 patients.

**Table 1:** Characteristics of the studies included in the systematic review.

N.	Author	Type of study	Study Region	N. of previous miscarriages	Mode of conception	LMWH				Control			
						N.	Age	BMI	Intervention	N.	Age	BMI	Intervention
1	Pasquier et al. (2015)^17^	RCT	Europe	2	Not specified	138	32.7 ± 5.2	23.9 ± 4.4	Enoxaparin 40 mg started before week 6 of gestation (or at least 1 week before the most advanced term reached before)	118	32.1 ± 5.4	23.9 ± 5	Placebo
2	Mohammad‑Akbariet al. (2023)^41^	RCT	Middle East	2	Spontaneous	82	30.13 (± 4.71)	25.51 (± 3.03)	Enoxaparin 40 mg and 80 mg of aspirin started after positive β -hCG test	83	29.68 (27–36)	26.4 (± 4.02)	80 mg of aspirin
3	Kaandorp et al. (2010)^21^	RCT	Europe	2	Not specified	97	34 ± 5	25.4 ± 4.9	Aspirin plus nadroparin (at a dose of 2850 IU) before week 6 of gestation	99	33 ± 5	25.0 ± 4.8	100 mg of aspirin placebo
										103	34 ± 5	24.6 ± 4.1	
4	Dolitzky et al. (2006)^19^	RCT	Middle East	3	Not specified	54	31.73 ± 5.3 (23–45)	NA	40 mg of enoxaparin from the time of detection of a fetal heart beat (6–12 weeks)	50	30.65 6.18(20–42)	NA	100 mg of aspirin
5	Giancotti et al. (2012)^29^	RCT	Europe	2	Not specified	25	NA	NA	Enoxaparin 40 mg (n = 25); enoxaparin 40 mg and LDA (n = 28); both after β-HCG and corresponding ultrasound scan	27	NA	NA	LDA 100 mg until third month of pregnancy
						28							
6	Visser et al. (2011)^30^	RCT	Europe	3	Spontaneous	68	32.5 ± 4.29	23.4 ± 3.71	Enoxaparin 40 mg (n = 68); enoxaparin and LDA (n = 63); both before week 7 of gestation	76	32.0 ± 4.47	25.4 ± 5.38	LDA 100 mg
						63	31.6 ± 4.57	23.1 ± 3.13					

### Risk of bias and quality assessment results

**Figure 2:**
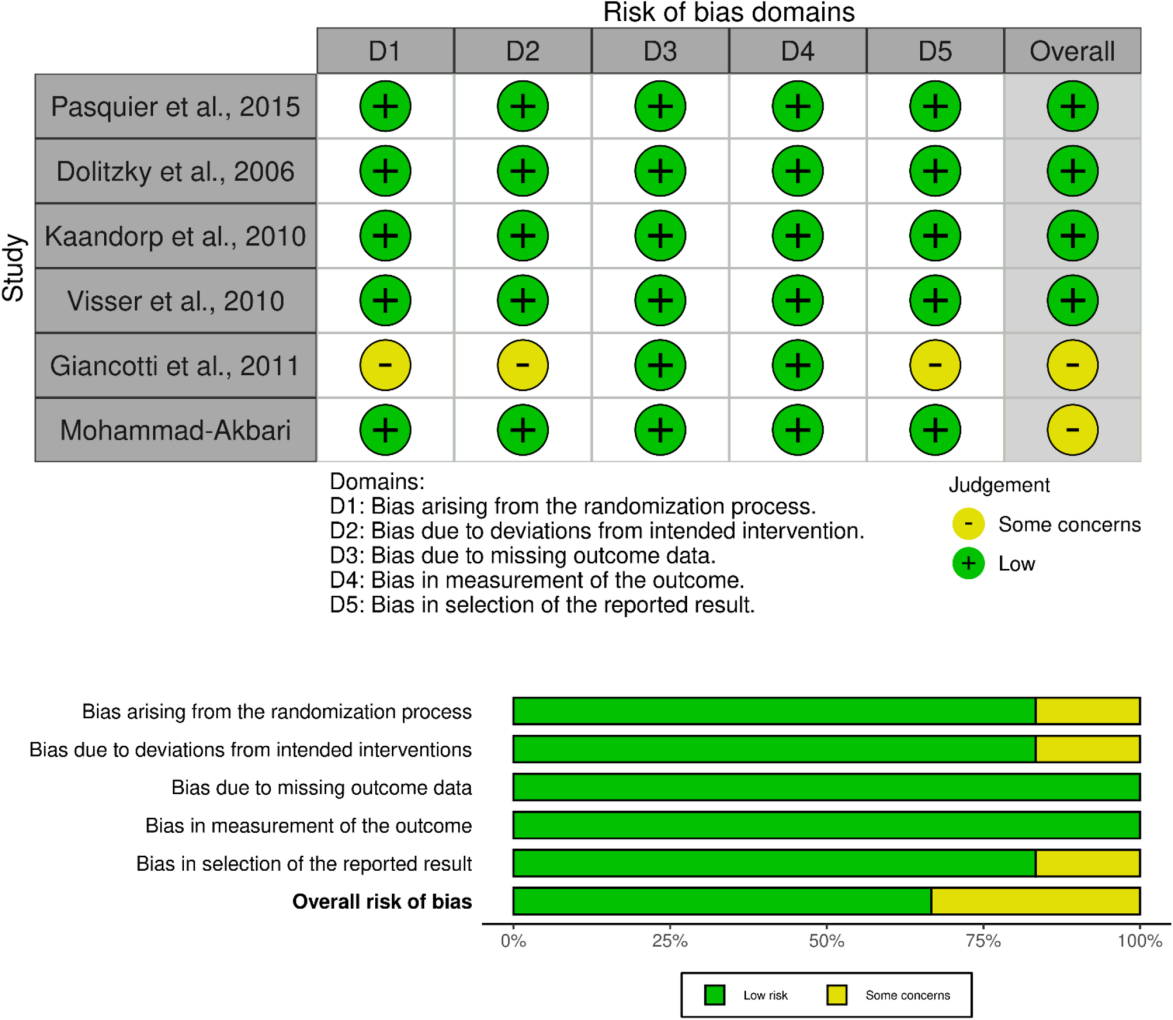
Assessment of risk of bias of randomized clinical trials included in this meta-analysis considering live-birth rate as outcome.

**Figure 3:**
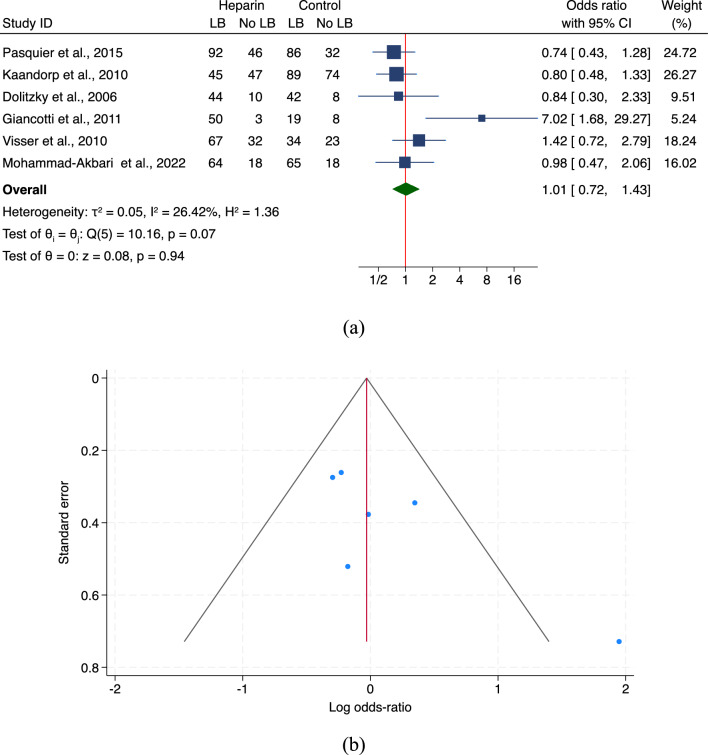
(**a,b**) Heparin and live births: random-effects meta-analysis of all included studies (n = 6). *LB* live births. (**a**) Forest plot: the estimate of the overall effect size is depicted by a green diamond centered at the estimate of the overall effect size. (**b**) Funnel plot for publication bias: Egger’s test: p = 0.0146; Begg’s test: p = 0.2597.

Results obtained from our risk of bias assessment for randomized clinical trials using RoB2 tool are summarized in Fig. 2. None of the included studies presented a high risk of bias in any of the five tool’s domains (randomization process, deviation from intended intervention, missing outcome data, measurement of the outcome, selection of the reported results). Visual inspection of funnel plots for the meta-analysis considering LBR suggests the presence of non-significant publication bias (Egger’s test for LBR: *p* = 0.0146, Fig. 3b).

A summary of quality of evidence assessment according to the GRADE system is reported in Table 2. Overall quality of evidence was moderate for RCTs, owing to the occurrence of bias in the randomization process, deviation from the intended intervention, lack of measurement of the outcome, poor ascertainment of exposure, and follow up not long enough. The TRACT analysis is presented in Table 3.

**Table 2:** Quality assessment of trials comparing LMWH versus controls divided for the main outcome (LBR).

	Quality assessment					Summary of findings					
	Design	Quality	Consistency	Directness	Other modifying factors	N. of patients		Effect			
						LMWH	Control	Relative (95% CI)	Absolute	Quality of evidence	Importance
LBR (n = 6)					None	518	498	OR 1.01 95% CI = 0.72, 1.43	No Difference	Moderate	Moderate
Pasquier et al. (2015)^17^	RCT	No serious limitations	No importan consistency	Direct							
Mohammad‑Akbariet al. (2023)^41^	RCT	No serious limitations	No importan consistency	Direct							
Kaandorp et al. (2010)^21^	RCT	No serious limitations	No importan consistency	Direct							
Dolitzky et al. (2006)^19^	RCT	Lack of power analysis	No importan consistency	Direct							
Giancotti et al. (2012)^29^	RCT	Lack of power analysis, randomization bias	Short Report	Direct							
Visser et al. (2011)^30^	RCT	No serious limitations	No importan consistency	Direct

**Table 3:** Quality assessment of trials included in the study according to the TRACT analysis.

N.	Author	Governance	Author group	Plausibility of intervention usage	Timeframe	Drop-out rates	Baseline characteristics	Outcomes
1	Pasquier et al. (2015)^17^	X	X	X	X	X	X	X
2	Mohammad‑Akbariet al. (2023)^41^	X	X	X	X	X	X	X
3	Kaandorp et al. (2010)^21^	X	X	X	X	X	X	X
4	Dolitzky et al. (2006)^19^	X	X	X	X	X	X	X
5	Giancotti et al. (2012)^29^	Absent	X	X	X	Concerns	X	Concerns
6	Visser et al. (2011)^30^	X	X	X	X	X	X	X

### Meta-analysis

#### Synthesis of results

The pooled risk estimates according to random effect models for the main primary outcome (LBR) are shown in Fig. 3a.

#### LMWH and LBR

A total of 6 studies were included in the quantitative synthesis of LMWH effect on LBR^17,19,21,29,30,41^. Results of the random-effects model according to DerSimonian–Laird method are showed in Fig. 3a,b. For all the included studies (*n* = 6) the pooled OR was equal to 1.01 (95% CI 0.72–1.43), with medium heterogeneity (I^2^ = 26.42%) and a non-significant publication bias (Egger’s test: *p* = 0.0146). Table 4 also shows results obtained applying a fixed-effects model (OR 0.98, 95% CI 0.75–1.30, I^2^ = 50.83%).

**Table 4:** Sub-group analysis of heparin administration and live births.

	N. studies	Effect estimates		Heterogeneity	*p-*value
		OR	[95% CI]	I^2^ (%)	
All included studies					
Random-effects	6	1.01	[0.72, 1.43]	26.42	0.94
Fixed-effects	6	0.98	[0.75, 1.30]	50.83	0.90
Study country					
Middle East	2	0.93	[0.51, 1.70]	0	0.82
Europe	4	1.28	[0.59, 2.76]	80.05	0.53
Diagnostic criteria					
≥ 3 early miscarriages	2	1.21	[0.69, 2.12]	0.00	0.51
≥ 2 early miscarriages	4	1.14	[0.55, 2.39]	77.02	0.72
Control type					
Placebo	2	0.32	[0.07, 1.55]	88.11	0.16
Aspirin	4	1.43	[0.74, 1.43]	53.25	0.29

*OR* odds ratio, *CI* confidence interval.

##### Subgroup analyses

In light of the high heterogeneity in the primary meta-analysis, subgroup analyses were performed according to study region (Middle East or Europe), diagnostic criteria for recurrent miscarriage (at least 2 or at least 3 early miscarriages), and control type (placebo or other treatments/none) (Table 4).

*Diagnostic criteria for U-RPL* According to diagnostic criteria for recurrent pregnancy loss, the pooled OR of studies defining recurrent miscarriage as at least 2 miscarriages (n = 4 studies) was not statically significant 1.14 (95% CI 0.55, 2.39), with *p* = 0.51, and carried a quite high heterogeneity (I^2^ = 77.02%). Studies defining unexplained recurrent pregnancy loss as at least 3 miscarriages (n = 2 studies) showed a non-significant pooled OR of 1.21 (95% CI 0.69, 2.12) with lower heterogeneity (I^2^ = 0.%).

*Study region* According to the study region, studies based in Middle East were two, with a pooled OR of 0.93 (95% CI 0.51, 1.70), and p = 0.82. On the contrary, the four European studies had a non-significant lower risk estimate (pooled OR 1.28; 95% CI 0.59, 2.76) with a high heterogeneity (I^2^ = 80.05%).

*Control type* Regarding the control type (placebo or other treatments/none) two studies administered placebo with a non-signficant OR of 0.32 (95% CI 0.07, 1.55), an high heterogeneity (I^2^ = 88.11%) and a p = 0.16. Similarly, the aspirin control group presented an OR of 1.43 (95% CI 0.74, 1.43), an high heterogeneity (I^2^ = 53.25%) and a p = 0.29.

## Discussion

The results of our meta-analysis show a non- significant effect of LMWH in U-RPL on LBR based on moderate quality registered RCTs. To the best of our knowledge, the present meta-analysis is the first on the use of LMWH in U-RPL to exclude retracted papers or papers with relevant integrity concerns^36^, and to provide subgroup analyses based on clinical features including number of previous miscarriages and control group. Compared to previous evidence in the field, our methodological approach thus allowed us to investigate the sources of heterogeneity, better explaining the conflicting existing literature. Altogether, our meta-analysis however does not support the use of LMWH in any subgroup of women with U-RPL. To mention, we strengthen the fact that our article did not include any thrombophilic condition, either acquired or congenital. This finding is in contrast with evidence from a previous meta-analysis on women with at least 3 or more previous abortion^31^, which however included non-registered RCTs as well as a subsequently retracted paper, and also with the most recent observational registry study on this subject, suggesting a beneficial effect of LMWH even in the absence of thrombophilia^42^. Of note, in such study the authors excluded patients with 2 previous miscarriages but without at least one documented previous euploid fetal karyotype. Abnormal embryonic karyotype in the index-pregnancy has a prevalence as high as 60% among couples with 2 previous miscarriages and might thus substantially confound the results of any RCT on the subject^43^. As a limitation of the existing RCTs and of the present meta-analysis, the absence of this relevant exclusion criteria might thus contribute to the discrepancy with the above-mentioned registry study^42^ and deserves further consideration (Supplementary Information).

Two further limitations of our work should also be considered. First, none of the available studies provides data on vaginal bleeding and/or on the use of progesterone, which could however confound the results given its beneficial role on LBR in women with one or more previous miscarriages^9,11^. Second, as we discussed previously^44^, the high prevalence of overweight women and the use of a standardized dose of LMWH not adjusted to body weight in the studies included might lead to an underdosage issue^45^ and confound the results. Lastly, other residual factors accounting for the heterogeneity of available studies remain, such as the different types and dosages of LMWH used. Indeed, different formulations of LMWH used (enoxaparin, nadroparin) may have different anticoagulant and anti-inflammatory properties. Nonetheless, a recent meta-analysis on LMWH in the prevention of preeclampsia and other placenta-mediated complications of pregnancy also found non significant differences in the type of heparin used^20^, partly reassuring about the generalizability of our results. Notably, this meta-analysis found that LMWH combined with LDA in high risk women reduced the risk of pre-eclampsia and other placenta-mediated complications compared to LDA alone—even in patients without thrombophilia, but not of miscarriage^20^. In addition, current literature does not allow to perform sub-analyses based on the mean of conception. Of the included studies, only two RCTs (Visser et al., Mohammad‑Akbariet et al.) excluded pregnancies obtained by assisted reproduction. Thus, whether recurrent miscarriage after assisted reproduction treatments (ART) and recurrent miscarriage after spontaneous conception might respond differently to LMWH prophylaxis thus remains unexplored. Altogether, current evidence thus discourages the use of LMWH in U-RPL, but highlights the need for further RCTs properly accounting for previous fetal karyotype, use of vaginal progesterone, patients’ BMI and mean of conception (spontaneous versus ART).

### Supplementary Information

Supplementary Information.

## Data Availability

Data are available upon reasonable request contacting the corresponding author.
